# Home Visits as a Primary Health Care Strategy During COVID-19: Protocol for a Scoping Review

**DOI:** 10.2196/97947

**Published:** 2026-08-10

**Authors:** Woska Pires da Costa, Heloísa de Carvalho Torres, Ana Paula Santana Coelho Almeida, Luiz Carlos de Abreu, Ana Cecília Oliveira Costa, Eugênia Aparecida Portes, Anny Tristão Vimercati, Vitória Andrade Rodrigues Moreira, Thiago Dias Sarti

**Affiliations:** 1 Research Department, Instituto Federal Goiano - Campus Morrinhos, BR-153 Highway, Km 633, P.O. Box 92, Rural Area, Morrinhos, GO, 75658-899, Brazil, 55 64992227424; 2 Medical School, Universidade de São Paulo (USP), São Paulo, SP, Brazil; 3 Nucleus for Education and Applied Research in Primary Health Care (NEAPS), Universidade Federal do Espírito Santo (UFES), Vitória, ES, Brazil; 4 Postgraduate Program in Collective Health, Universidade Federal do Espírito Santo (UFES), Vitória, ES, Brazil; 5 Postgraduate Program in Nutrition and Health, Universidade Federal do Espírito Santo (UFES), Vitória, ES, Brazil; 6 Department of Rehabilitation Sciences, Florida Gulf Coast University (FGCU), Fort Myers, FL, United States

**Keywords:** primary health strategy, family health teams, home-based care, community health services, public health system, public health emergencies, COVID-19, scoping review

## Abstract

**Background:**

Primary health care played a critical role during the COVID-19 pandemic by adapting care delivery to maintain essential services and reduce transmission risks. Home visits were used to monitor individuals in isolation, support vulnerable populations at increased risk, and sustain community-based care. However, their organization and operationalization varied across settings, and the available evidence remains fragmented.

**Objective:**

This protocol outlines the methods for a scoping review that will systematically map how in-person or hybrid home visits linked to primary, community-based, or first-contact care were organized and operationalized during the COVID-19 response, including the operational approaches, reported outcomes, and remaining knowledge gaps.

**Methods:**

This protocol follows the Joanna Briggs Institute methodological framework and will be reported in accordance with the PRISMA-ScR (Preferred Reporting Items for Systematic Reviews and Meta-Analyses Extension for Scoping Reviews) statement. Eligibility was defined using the Population, Concept, and Context framework. Searches will be conducted in seven electronic databases (Scopus, Web of Science Core Collection, MEDLINE/PubMed, Embase, Cochrane Library, CINAHL, and LILACS) without language restrictions and will include eligible studies published from 2020 onward. Conditional supplementary procedures may include backward citation searching and citation mapping of included studies and relevant reviews, as well as targeted searches and limited gray literature searching, if database searches yield limited evidence or leave relevant questions insufficiently represented. Records will be managed in Rayyan. Two reviewers will independently assess eligibility, with disagreements resolved by a third reviewer. All included sources will undergo descriptive mapping. Operational configurations will be classified by provider configuration, delivery modality, and targeting logic. Basic qualitative content analysis will be limited to explicitly reported implementation experiences, barriers, facilitators, contextual adaptations, and recommendations.

**Results:**

Pilot searches were conducted solely to assess feasibility and refine the search strategy; formal searching, screening, data extraction, and synthesis have not yet begun. The review will commence after acceptance of the protocol and is expected to be completed within approximately 6 months.

**Conclusions:**

The review will provide a structured evidence map of home-visit practices during the COVID-19 response and may identify transferable operational lessons and evidence gaps relevant to preparedness for future public health emergencies. Reported outcomes will not be interpreted as evidence of comparative effectiveness or causal effects.

## Introduction

### Background

Primary health care (PHC) is a cornerstone of equitable, accessible, and comprehensive health systems and plays a central role in advancing universal health coverage and strengthening health system resilience [1-3]. By providing first-contact, continuous, coordinated, and community-oriented care, PHC supports the delivery of essential services across the life course. Its role is particularly important in low- and middle-income countries, where primary care often serves as the primary point of contact between individuals, communities, and the health system [4-6].

The capacity of PHC systems to maintain essential services, coordinate community-based responses, and adapt care delivery becomes especially important during public health emergencies [2,5,7]. During the COVID-19 pandemic, PHC services underwent rapid and substantial reorganization to respond simultaneously to SARS-CoV-2 transmission and continuing population health needs. These changes included expanded remote support, modified care pathways, prioritization of populations at increased risk, strengthened surveillance activities, participation in vaccination campaigns, and responses to emerging clinical and social needs [8-12].

These adaptations occurred under considerable structural and operational constraints. Health services faced workforce overload, shortages of personal protective equipment (PPE), disruption of routine care, fragmented coordination between PHC and public health surveillance, and inequalities in access to digital technologies [5,10,13-16]. The nature and intensity of these challenges varied across countries, health systems, socioeconomic settings, and phases of the pandemic, influencing how primary care teams organized and delivered community-based services.

Within this broader reorganization of PHC, home visits represented an important strategy for maintaining care outside health facilities. Traditionally, home visits support longitudinal and person-centered care by enabling health professionals to assess clinical, social, environmental, and family needs within individuals’ living environments [17-20]. They may encompass clinical assessment and follow-up, health promotion, disease prevention, rehabilitation, health education, psychosocial support, health surveillance, medication or supply delivery, and care coordination. Home visits can also strengthen relationships among health services, individuals, families, and communities, particularly in territorially organized or community-oriented models of primary care.

During the COVID-19 response, the organization and purposes of home visits were adapted to pandemic-related conditions. These visits were used to monitor individuals in isolation, support vulnerable or high-risk populations, maintain care for chronic conditions, deliver medications or essential supplies, undertake surveillance activities, and respond to emerging clinical and social needs [9]. In some settings, home visits were incorporated into hybrid models that combined direct in-person contact in the home with telephone follow-up, video consultations, remote triage, or digital monitoring. Community health workers, nurses, physicians, multidisciplinary teams, and dedicated outreach personnel assumed distinct roles in care coordination, risk assessment, community engagement, health communication, and the maintenance of territorial links [5,16].

The operationalization of these strategies was influenced by epidemiological conditions, vaccine availability, diagnostic capacity, public health restrictions, workforce organization, availability of PPE, digital infrastructure, and local models of care [8,11,13,14,16]. Consequently, home-visit strategies differed in their provider configurations, delivery modalities, purposes, prioritization criteria, infection prevention and control procedures, levels of digital integration, and relationships with other primary and community-based services. International organizations emphasized the strengthening of community-based care and telehealth capacity as components of preparedness and response to public health emergencies [21]. Nevertheless, differences between recommended practices and their implementation in real-world settings remain insufficiently mapped, and changes in primary care organization and use were not uniform across health systems [8,12].

Evidence on home visits during the COVID-19 response remains dispersed across countries, disciplines, professional groups, and service settings and is reported using inconsistent terminology. Although individual studies describe changes in the frequency, purposes, organization, and delivery of home visits, there is limited systematization of how in-person and hybrid home visits linked to primary, community-based, or first-contact care were organized and operationalized. This fragmentation limits the identification of recurrent operational configurations, context-sensitive adaptations, implementation barriers and facilitators, reported process or service outcomes, and transferable lessons for future public health emergencies. A focused scoping review is therefore warranted to map the breadth, characteristics, and distribution of this evidence without presuming comparative effectiveness or causal impact.

### Search for Existing Reviews and Protocols

Preliminary searches of review registries and bibliographic databases conducted on November 10, 2025, did not identify a registered or published review specifically mapping the organization and operationalization of home visits linked to primary care during the COVID-19 response. This gap supports a focused scoping review designed to map implementation characteristics and contextual variation rather than to estimate effectiveness.

### Objective and Review Questions

The objective of this scoping review is to systematically map and descriptively synthesize the available evidence on how in-person or hybrid home visits linked to primary, community-based, or first-contact care were organized and operationalized during the COVID-19 response. The review will describe provider configurations, delivery modalities, visit purposes, prioritization criteria, infection prevention and control procedures, levels of digital integration, and temporal or contextual adaptations in relation to implementation periods and locally reported pandemic milestones. It will also map explicitly reported implementation barriers, facilitators, contextual influences, process or service outcomes, and remaining knowledge gaps across different health system settings.

To address this objective, the following research questions (RQs) were formulated:

RQ1: Which provider configurations, delivery modalities, purposes, prioritization criteria, safety procedures, levels of digital integration, and temporal or contextual adaptations were reported?
RQ2: Which barriers, facilitators, and contextual factors influenced the implementation and adaptation of these home-visit strategies?
RQ3: Which process or service outcomes and remaining knowledge gaps were reported?

## Methods

### Methodological Framework

This scoping review will be conducted in accordance with the methodological framework proposed by the Joanna Briggs Institute for scoping reviews [22-24], complemented by the foundational framework established by Arksey and O'Malley [25]. The completed review will be reported according to the PRISMA-ScR (Preferred Reporting Items for Systematic Reviews and Meta-Analyses Extension for Scoping Reviews) [26]. This protocol was developed using the PRISMA-P (Preferred Reporting Items for Systematic Review and Meta-Analysis Protocols) 2015 checklist [27,28] (Checklist 1), and the methodological procedures were predefined in line with established evidence-synthesis practices [29]. Scoping reviews are particularly appropriate for mapping heterogeneous evidence, identifying knowledge gaps, and clarifying key concepts in complex and rapidly evolving fields [30,31].

### Ethical Considerations

This scoping review protocol was prospectively registered in the Open Science Framework Registries [22,29,32,33]. Any modifications to the protocol during the review will be documented, updated in the registry, and explicitly reported in the final publication, ensuring full transparency and methodological accountability [34]. Because the study will use only previously published sources and will not involve direct interaction with human participants, ethical approval is not required [35,36].

### Eligibility Framework and Definitions

The review question was formulated based on the PCC (Population, Concept, and Context) framework to predetermine eligibility criteria and guide the identification of relevant studies, in line with scoping review methodological guidance [22,23]. This framework was used to define the principal elements of the research focus and establish transparent and reproducible eligibility boundaries [37]. The PCC framework ensures conceptual coherence between the review question, eligibility criteria, study selection, and data extraction. However, it does not require every PCC component to be represented as a mandatory block in the database search strategy. In the present review, the Population component will primarily guide eligibility assessment during screening because primary care affiliation and provider type may be inconsistently reported in titles, abstracts, keywords, and indexing fields.

This structured approach is particularly appropriate for investigating complex health interventions influenced by multiple interacting professional, organizational, territorial, and epidemiological factors. The review will therefore focus on home visits that involve an in-person component and are delivered by professionals or teams responsible for, formally linked to, coordinated with, or referred through primary, community-based, or first-contact care.

The primary review question is, “How were in-person or hybrid home visits linked to primary, community-based, or first-contact care organized and operationalized during the COVID-19 response?” The PCC framework and the operational definitions that will guide eligibility for the review are presented in Table 1.

**Table 1.** PCC (Population, Concept, and Context) framework^a^.

Component	Definition
P (Population)	Health professionals, community health workers, outreach personnel, or multidisciplinary teams responsible for or formally linked to primary, community-based, or first-contact care, irrespective of the terminology used to describe the service in each country or health system. Eligible providers may include family physicians, general practitioners, nurses, community health workers, allied health professionals, and dedicated outreach personnel.
C (Concept)	Home visits are defined as in-person encounters conducted in an individual’s or family’s place of residence. They may involve clinical assessment, monitoring, treatment, health surveillance, health education, psychosocial support, medication or supply delivery, preventive care, rehabilitation, or care coordination [17,19,20]. Hybrid models combining an in-person home component with telephone, video, digital monitoring, or other remote support will be eligible [8,12,21]. Contacts conducted exclusively through telephone, video, mobile applications, or other remote technologies, without an in-person encounter in the home, will not be classified as home visits.
C (Context)	The review will consider home visits integrated with, coordinated by, or referred through primary, community-based, or first-contact care in any country or health system during the COVID-19 response. This context encompasses the period beginning in 2020 during which health services were reorganized in response to SARS-CoV-2 transmission, epidemiological waves, public health restrictions, vaccination rollout, and the progressive restoration of routine services. The World Health Organization declared COVID-19 a pandemic on March 11, 2020 [38,39]. Within primary and community-based care, the pandemic led to changes in service delivery, surveillance practices, infection-control procedures, prioritization criteria, workforce organization, and the integration of in-person and remote care, directly affecting the organization and delivery of home visits [8-12,40].

a PCC is a strategy used to aid in scoping reviews that defines the key elements of research: P delineates the Population, C specifies the Concept, and the second C details the Context. To maximize search sensitivity, the core database search will combine terms related to the Concept and Context, whereas the Population component and linkage to primary, community-based, or first-contact care will be assessed during study screening and confirmed through full-text review.

### Information Sources

To ensure a comprehensive, systematic, and internationally relevant evidence base, we selected databases with broad coverage, multidisciplinary scope, and relevance to the topic under investigation [41]. This scoping review will consider evidence from studies using quantitative, qualitative, and mixed methods designs, reflecting the methodological diversity expected in the field [42]. The review question and the predefined eligibility criteria guided the selection of information sources.

To identify relevant literature, searches will be conducted in the following seven electronic databases: Scopus, Web of Science Core Collection, MEDLINE/PubMed via the National Library of Medicine interface, Embase, Cochrane Library, CINAHL (CINAHL Plus) via EBSCOhost, and Latin American and Caribbean Health Sciences Literature (Literatura Latino-Americana e do Caribe em Ciências da Saúde; LILACS) via the Virtual Health Library (Biblioteca Virtual em Saúde) interface. These databases were selected because they collectively provide extensive international coverage across biomedical, public health, and interdisciplinary research domains, thereby maximizing the sensitivity and comprehensiveness of the search and reducing the risk of publication bias.

### Search Strategy

A predefined and structured search strategy will be used to identify studies aligned with the objectives of this scoping review [43]. The strategy was developed iteratively, incorporating both controlled vocabulary (eg, MeSH and Emtree terms) and free-text keywords to enhance sensitivity and specificity. The research team will adapt the search syntax for each database by incorporating the relevant controlled vocabulary and free-text terms identified for the review topic. The search syntax will be adapted for each database according to its indexing system, search interface, controlled vocabulary, field codes, and syntax requirements (Multimedia Appendix 1).

Although the PCC framework guides the eligibility criteria, terms related to PHC affiliation, professional categories, or team composition will not constitute a mandatory block in the core search strategy. Because these characteristics may be inconsistently reported in titles, abstracts, keywords, and indexing fields, requiring such terms could reduce search sensitivity and exclude potentially eligible records. Linkage to primary, community-based, or first-contact care, as well as provider type, will therefore be assessed during screening and confirmed through full-text review.

Accordingly, the principal search strategy will combine two conceptual blocks: the home-visit concept and the COVID-19 context. Within each block, synonyms, controlled descriptors, and related free-text terms will be combined using the Boolean operator “OR,” whereas these conceptual blocks will be combined using the Boolean operator “AND” [41]. The relationship of each record to primary, community-based, or first-contact care will be assessed during title and abstract screening and confirmed through full-text review. This approach prioritizes search sensitivity while preserving the review’s predefined eligibility boundaries. The conceptual structure of the search strategy is presented in Table 2.

**Table 2.** Conceptual blocks used in the core database search^a^.

Blocks (PCC)^b^	Keywords used
#1 (Concept) Home-visit concept	(“home visit*” OR “house call*” OR “home care service*” OR “home health care” OR “home nursing” OR “home-based care” OR “domiciliary visit*” OR “domiciliary care” OR “home-based primary care” OR “community home visiting” OR “household visit*” OR “home follow-up” OR “in-home care” OR “in-home health service*” OR “outreach visit*” OR “family health strategy” OR “remote patient monitoring”)
#2 (Context) COVID-19	(“COVID-19” OR “COVID 19” OR “COVID19” OR “COVID-2019” OR “SARS-CoV-2” OR “SARS CoV 2” OR “SARS-CoV2” OR "2019-nCoV” OR “nCoV-2019” OR “SARS coronavirus 2” OR “severe acute respiratory syndrome coronavirus 2” OR “novel coronavirus” OR “coronavirus infection*” OR “coronavirus pandemic” OR “coronavirus disease”)
Search string	(#1) AND (#2)

a PCC is a strategy used to aid in scoping the reviews that defines the key elements of research: P delineates the Population, C specifies the Concept, and the second C details the Context. To maximize search sensitivity, the core database search will combine terms related to the Concept and Context, whereas the Population component and linkage to primary, community-based, or first-contact care will be assessed during study screening and confirmed through full-text review.

b PCC: Population, Concept, and Context.

A preliminary pilot search was conducted exclusively to assess the volume and nature of the records retrieved and to identify any adjustments needed to improve the strategy’s sensitivity and precision. This pilot phase enabled the refinement of keywords, the identification of additional relevant terms, and the optimization of search performance. No formal study selection, data extraction, or analysis was carried out at this stage. All full searches and subsequent review procedures described in this protocol will be conducted only after the acceptance of this manuscript. This approach ensures methodological integrity and avoids potential bias arising from premature screening or analysis.

The complete search strategy was developed in accordance with the PRESS (Peer Review of Electronic Search Strategies) 2015 recommendations [44] and will be reported according to PRISMA for Searches (PRISMA-S) recommendations [45]. Accordingly, any refinement filters applied during the searches will be fully documented, and the complete database-specific strategies will be presented in Multimedia Appendix 1. In addition, term-level return tests were conducted across all databases to assess the relevance of each search term, refine the strategy, estimate the expected retrieval volume, and evaluate the review’s feasibility (Multimedia Appendix 2).

### Secondary Sources

If the database searches yield a limited number of eligible studies (eg, fewer than 10 studies after full-text assessment), supplementary search procedures may be undertaken to enhance the comprehensiveness of the evidence base. These procedures may include targeted searches for government reports, institutional documents, official guidelines, and public policy documents [46]; a limited complementary gray literature search (eg, screening the first 100 Google Scholar results ranked by relevance), where appropriate [43]; backward citation searching of the reference lists of included studies and relevant reviews; and citation mapping (eg, using the Litmaps platform) [46-48].

If these procedures are conducted, the search sources, complete search terms or queries, search dates, sorting or ranking methods, number of results or pages examined, and number of records screened will be documented in the final review to ensure transparency and reproducibility. Where Research Information Systems format export is available, the corresponding metadata files will be provided as supplementary material to support the traceability and verification of the search process. All records identified through supplementary searches will undergo the same eligibility assessment and study selection procedures as records retrieved from the bibliographic databases.

Dissertations, theses, and undergraduate final course papers returned in the search procedures will not be included in the scope of this review [43], nor will other documents lacking identifiable authorship, methodological transparency, verifiable provenance, or recognized institutional authority. This restriction is intended to maintain consistency in the level of evidence and ensure alignment with accepted peer-reviewed scientific standards.

### Metadata Extraction

Metadata retrieved from the scientific databases will be exported at a single time point when the formal review begins [43,46], following the acceptance of this protocol. The exact search and export dates will be documented for each source. The initial forecast is that this stage will take place in August 2026; if necessary, the schedule may be adjusted according to the duration of the editorial process. Raw metadata files will be deposited in an open-access repository linked to the final review manuscript, supporting transparency and reproducibility [49], and future updating of the review [43,46].

### Eligibility Criteria

This scoping review will include studies without language restrictions and with various methodological designs (qualitative, quantitative, or mixed methods), published between January 1, 2020, and the date of metadata extraction, provided they meet all inclusion criteria and do not fall under any previously defined exclusion criteria. The adopted time frame is justified by the emergence of the COVID-19 pandemic, which began in Wuhan, China, in December 2019 [50,51].

Inclusion criteria are as follows:

- Peer-reviewed journal articles reporting original studies [36,52] or, if the secondary search is undertaken, authoritative governmental or institutional documents describing implemented practices, operational guidance, or policy measures directly relevant to the review question are included.
- Sources published from January 1, 2020, to the date of the final search are included.
- Sources reporting in-person or hybrid home visits delivered by professionals or teams responsible for, formally linked to, coordinated with, or referred through primary, community-based, or first-contact care during the COVID-19 response are included.

Exclusion criteria are as follows:

- Duplicate records from different databases will be identified using Rayyan’s “Auto Resolver” feature, applying a similarity threshold of at least 95% [49], followed by manual verification. Any remaining duplicates will be assessed and removed using the Bramer method [53].
- Dissertations, theses, or undergraduate monographs are excluded. Review studies addressing the topic will not be included in this review; however, they will be separated for use in the secondary stage of citation mapping [46]. The reference lists of these reviews will be examined, and if they include studies within this review’s scope, those studies may be included in the analysis.
- Studies that are not fully available in the databases consulted and cannot be obtained even after attempts to contact the authors are excluded [36,52,54].
- Articles written in languages for which an adequate translation is not feasible are excluded [55]. This criterion will only be applied after exhausting all translation possibilities, including (1) seeking support from the international network of collaborators; (2) using AI tools for preliminary comprehension; and (3) hiring specialized professional translation services [41]. No eligibility decision will be based solely on unverified machine-translated content; any translated text used to support an eligibility decision will be verified by a multilingual collaborator or professional translator. This translation tool, language, purpose, and verification procedure will be transparently reported in the final review.
- Studies with retraction records are excluded [55].

### Metadata Management

Reference management and study screening will be conducted using the Rayyan software (Rayyan Systems Inc), a widely used platform for systematic and scoping reviews that enhances efficiency, organization, and transparency in study selection [35,56]. This tool enables researchers to indicate the reasons for including or excluding studies based on predefined eligibility criteria, add tags to justify their decisions, and identify relevant terms, while also offering visual and filtering features that facilitate the screening process [57].

Rayyan will also be used for duplicate identification, blinded screening, labeling, and conflict resolution. The AI-assisted relevance ranking or automated exclusion features will not be used to determine eligibility. Each record will be assessed independently by 2 human reviewers, and disagreements will be resolved by a third senior reviewer. All inclusion and exclusion decisions will be made by human reviewers, ensuring adherence to methodological standards and minimizing the risk of automation bias.

### Reviewer Training

A pilot assessment will be conducted to train reviewers under the guidance of an experienced researcher, to standardize screening decisions and ensure consistent application of the predefined eligibility criteria [46,58]. This training phase is essential for enhancing interreviewer reliability and reducing potential inconsistencies during the study selection process. The training will also include familiarization with Rayyan and its functionalities [46,57], such as label use, filtering tools, and conflict-resolution features, thereby promoting uniformity in the review process and reducing potential disagreements among reviewers. If necessary, calibration exercises will be repeated until an acceptable level of agreement between reviewers is achieved.

### Selection Process

The review process will only begin after all search results have been imported into Rayyan, following these steps [49,58]: (1) removal of duplicates (first automatically for similarity ≥95%, followed by manual verification, when necessary); (2) screening of titles and abstracts by 2 independent reviewers; (3) assessment of agreement between reviewers; and (4) full-text assessment for the final inclusion decision. During screening, Rayyan labels and filtering features will be used to support reviewer decisions and to identify records that do not meet the previously established eligibility criteria [47].

A third senior researcher with expertise in the review topic will resolve any disagreements between reviewers [59,60]. This arbitration process ensures consistency and methodological rigor in inclusion decisions. The coauthors of this study will act as reviewers, and additional reviewers may be included if necessary to ensure the feasibility and timely completion of the review [61]. When the full text of a study is not available, the corresponding author will be contacted to request the article and, if relevant, the associated dataset [32]. Finally, each record will be classified as “included” or “excluded” according to the predefined criteria [47].

All steps will be documented in a flowchart in accordance with reporting guidance for scoping reviews, covering the selection of information sources, screening, recursive citation tracking, and final study inclusion [26,34]. All decisions made will be recorded, ensuring transparency, reproducibility, and completeness throughout the process [43]. Systematic searches of scientific databases will constitute the mandatory primary pathway for evidence identification [46]. Conditional secondary procedures may include backward citation searching and citation mapping of included studies and identified reviews, as well as targeted gray literature searches for pertinent governmental or institutional documents. Records identified through either pathway will undergo the same eligibility assessment and study selection procedures. These complementary approaches are intended to enhance the comprehensive analysis of the topic.

### Data Extraction

Data will be extracted regarding the publication (title, year, and DOI), the research context (country, institution, study setting, implementation period, scope, and population studied or participant groups), the methodological design (study design, analytical approach, and sample size), and the main findings of each study included in the review [41]. In addition, specific variables related to the organization, implementation, and reported process or service outcomes of home visits will be extracted, in accordance with the objectives and review questions.

Where reported, data will be extracted on the month and year of implementation, country or region, local vaccine availability, prevailing service restrictions, relevant epidemic waves or variants, testing capacity, remote triage, and restoration of routine services. Fixed calendar-year cutoffs and universal pandemic-phase categories will not be applied because these transitions occurred at different times across countries, regions, and health systems. When included sources explicitly define pandemic periods or phases, the classifications reported by their authors will be retained and described.

An extraction form (Multimedia Appendix 3) will be used to organize the data, allowing systematic synthesis and a structured description of the characteristics of the included studies [62]. The form will be reviewed and pilot-tested by the research team using a sample of eligible studies and refined, if necessary, before full data extraction. Any modifications made during this process will be documented. The use of a standardized extraction form enhances consistency, reduces extraction bias, and facilitates comparability across studies.

### Methodological Quality and Risk of Bias Assessment

The methodological quality and risk of bias of the included studies will not be formally assessed in this scoping review. This approach is consistent with scoping review methodology, which aims to map and describe the extent and characteristics of the available evidence rather than appraise study quality or establish causal inferences [23,26]. Accordingly, the inclusion of studies will be based solely on the predefined eligibility criteria, regardless of their methodological quality.

### Data Analysis and Synthesis

All included studies will first undergo descriptive mapping. Study characteristics—including publication year, country or region, study design or document type, participant groups, provider configurations, delivery modalities, visit purposes, prioritization criteria, safety measures, levels of digital integration, temporal context, and reported process or service outcomes—will be summarized using frequencies, tables, figures, and narrative descriptions. This mapping will provide an overview of the extent, distribution, and characteristics of the available evidence.

Operational configurations will be coded across three non–mutually exclusive dimensions: (1) provider configuration, including single-discipline, multidisciplinary or interprofessional, community health worker-led, and dedicated outreach arrangements; (2) delivery modality, classified as exclusively in-person or hybrid, with hybrid components further described as synchronous remote contact, asynchronous communication, or digital monitoring; and (3) targeting logic, including proactive risk-stratified surveillance, scheduled continuity or follow-up care, reactive responses to acute symptoms, and surveillance or isolation support. The combination of these dimensions will characterize each operational configuration. The categories may be refined during pilot testing, and all refinements and decision rules will be documented before full data extraction.

Temporal and operational changes will be described using the implementation period and locally reported contextual milestones, such as vaccine availability, epidemic waves, testing capacity, service restrictions, remote triage, and restoration of routine services. When included sources explicitly define pandemic periods or phases, the classifications reported by their authors will be retained. Sources that do not provide sufficient contextual information will not be retrospectively assigned to a predefined pandemic phase.

Basic qualitative content analysis will be limited to sources that explicitly report textual information on implementation experiences, barriers, facilitators, contextual adaptations, or recommendations. Coding will remain closely grounded in the manifest content of the source material and will combine categories predefined in the data extraction framework with additional categories developed inductively, where necessary [63]. The analytical process will be supported by NVivo software (QSR International Pty Ltd), which will be used solely to organize, code, and retrieve the relevant qualitative data [61,64]. No meta-aggregation, meta-ethnography, or higher-order interpretive thematic synthesis will be undertaken.

Quantitative information will be summarized using frequencies and other appropriate descriptive statistics, where applicable. Given the anticipated heterogeneity of study designs, contexts, interventions, and reported outcomes, no meta-analysis will be conducted.

The results will be synthesized through a structured narrative synthesis supported by tables and figures. The synthesis will describe how in-person or hybrid home visits linked to primary, community-based, or first-contact care were organized and operationalized during the COVID-19 response, highlighting similarities and differences across countries, health systems, socioeconomic contexts, and locally reported temporal periods. Particular attention will be given to provider configurations, delivery modalities, targeting and prioritization criteria, safety procedures, digital integration, contextual influences, implementation barriers and facilitators, reported process or service outcomes, and remaining knowledge gaps. Outcomes will be mapped as reported in the included sources and will not be interpreted as evidence of comparative effectiveness or causal effects.

### Reporting and Dissemination

The study selection process and findings will be reported using a combination of textual descriptions, tables, and visual elements to ensure clear alignment between the results, the review objectives, and the RQs [42]. The presentation of results will be guided by the PCC framework, supporting the structured organization of evidence according to the key components of the review [23]. Extracted data will be systematically organized in spreadsheets and provided as supplementary material to enhance transparency and reproducibility.

The evidence gathered may be illustrated through figures, diagrams, or other visual resources to clearly represent the organization of factors, as well as emerging patterns and trends [65,66]. These visual and structured reporting strategies will facilitate the interpretation of the findings, support knowledge translation, and enhance the usability of the results for researchers, practitioners, and policymakers.

## Results

The protocol was prospectively registered in the Open Science Framework on November 22, 2025. Preliminary pilot searches were conducted on June 22, 2026, solely to assess feasibility, examine the nature and volume of retrieved records, and refine the search strategy. No formal study selection, data extraction, or evidence synthesis was undertaken during this stage.

The formal review will commence after acceptance of the protocol, with the exact start date depending on the duration of the editorial process (it is expected to begin in August 2026). Database searching, metadata export, duplicate removal, screening, data extraction, and evidence synthesis are expected to be completed within approximately 6 months of formal initiation, with completion anticipated by February 2027.

**Figure 1.**
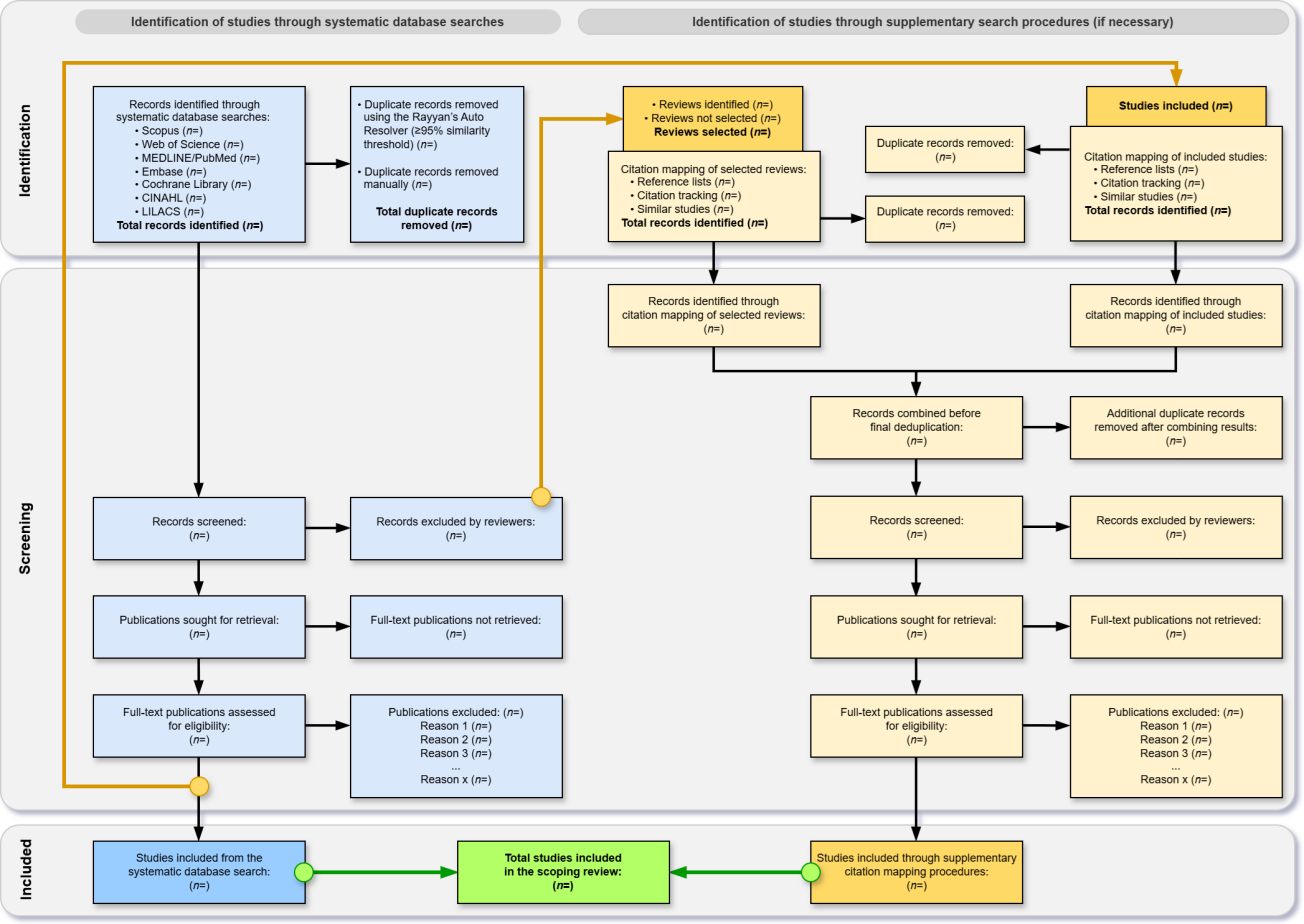
PRISMA (Prospective Preferred Reporting Items for Systematic Reviews and Meta-Analyses) 2020 flow diagram for the study selection process. The primary pathway (shown in blue) is mandatory. The screening process and full-text eligibility assessment within this pathway will be conducted independently by 2 reviewers. The secondary pathway (shown in yellow) is optional and conditional and will be used only if the primary pathway yields a limited number of eligible studies or leaves relevant aspects of the review questions insufficiently represented. The green box represents the final set of studies included through the primary pathway and, when applicable, the secondary pathway.

The prospective PRISMA-based study selection flow diagram will be used to document database identification, duplicate removal, title and abstract screening, full-text assessment, citation tracking, and final inclusion (Figure 1). Because the formal review has not yet begun, numerical counts are not reported at the protocol stage. The completed diagram will be included in the final scoping review.

## Discussion

### Expected Contributions and Interpretation

The planned review is expected to provide a structured evidence map of the operational configurations used to deliver in-person and hybrid home visits during the COVID-19 response. Its principal contribution will be to organize fragmented evidence on provider arrangements, delivery modalities, targeting strategies, safety procedures, digital integration, and context-sensitive adaptations across different health systems. Rather than determining which configuration was most effective, the review will clarify how these strategies were implemented and which operational lessons and evidence gaps may be relevant to future public health emergencies.

Although home visits are a well-established component of PHC, their delivery was substantially reconfigured across health systems to maintain continuity of care under conditions of social distancing and heightened infection risk [8,9,11,67,68]. The review will examine variations across countries, health systems, socioeconomic contexts, implementation periods, and locally reported pandemic milestones in provider configurations, delivery modalities, visit purposes, prioritization criteria, infection prevention and control procedures, digital integration, and other contextual adaptations. These variations will be interpreted as differences in implementation and context rather than as evidence that one model is more effective than another.

The evidence map will also describe the barriers, facilitators, and contextual factors that influenced the implementation and adaptation of home-visit strategies. Particular attention will be given to the roles of community health workers and multidisciplinary teams in care coordination, community engagement, and the maintenance of territorial links, which have been recognized as important but remain insufficiently explored or inconsistently reported in empirical studies [10,16]. Reported process and service outcomes may include continuity of care, access to services, patient and community engagement, clinical monitoring, psychosocial support, medication or supply delivery, and care coordination. These outcomes will be mapped according to how they are reported in the included studies and will not be interpreted as evidence of comparative effectiveness or causal impact. This approach is consistent with the purpose of a scoping review, which is to characterize the breadth, nature, and distribution of the available evidence rather than determine intervention effectiveness.

Anticipated challenges include workforce overload, shortages of PPE, resource constraints, technological and logistical barriers, territorial inequalities, heterogeneous terminology, variation in health system organization, and inconsistent reporting. Although these factors may limit direct comparisons, mapping them may support the identification of context-sensitive lessons for strengthening PHC workforce preparedness and home-based care strategies in future public health emergencies.

### Future Directions and Dissemination

The review is expected to identify areas in which operational guidance, empirical evaluation, and comparative research remain limited. These gaps may inform the development of future protocols, implementation studies, and context-sensitive guidance for home-based care during public health emergencies. Findings will be disseminated through a peer-reviewed publication, scientific meetings, and, where appropriate, concise materials for health professionals, service managers, and policymakers.

### Strengths and Limitations

Among the anticipated strengths of this review, we highlight the methodological rigor grounded in internationally recognized scoping review guidelines, the inclusion of multiple scientific databases and potential complementary searches of gray literature when needed, which enhance search sensitivity and increase the likelihood of identifying diverse international perspectives and experiences. The use of a standardized extraction form, a transparent search strategy, descriptive mapping, and targeted basic qualitative content analysis will further support the consistency and reproducibility of the review findings.

However, some limitations should be acknowledged. The heterogeneity of study designs and variability in terminology used to describe home visits may limit comparability across studies. Furthermore, differences in health system organization and reporting practices across countries may introduce contextual variability that affects the interpretation and generalizability of the findings. In addition, as no formal assessment of methodological quality will be conducted, the findings will not support conclusions regarding effectiveness or causal inference. Finally, the unequal geographic distribution of the available literature may introduce regional biases in the representation of the results. Despite these limitations, the scoping review design is appropriate for mapping and synthesizing the breadth of available evidence in this field.

### Conclusion

This protocol establishes a focused and reproducible approach to mapping the organization and operationalization of home visits linked to primary care during the COVID-19 response. By separating provider typology from the place of care, applying a multidimensional implementation framework, and examining temporal variation, the review is positioned to identify transferable operational lessons and priority evidence gaps without presuming effectiveness.

## Supplementary material

10.2196/97947 Multimedia Appendix 1 Details of the Boolean search string used for each database.

10.2196/97947 Multimedia Appendix 2 Return tests for the terms selected for the mandatory primary search pathway.

10.2196/97947 Multimedia Appendix 3 Data extraction summary form of the studies included in this scoping review.

10.2196/97947 Checklist 1 PRISMA-P checklist for the scoping review protocol.

## Abbreviations

LILACS: Latin American and Caribbean Health Sciences Literature
PCC: Population, Concept, Context
PHC: primary health care
PPE: personal protective equipment
PRESS: Peer Review of Electronic Search Strategies
PRISMA-P: Preferred Reporting Items for Systematic Review and Meta-Analysis Protocols
PRISMA-S: Preferred Reporting Items for Systematic Reviews and Meta-Analyses for Searching
PRISMA-ScR: Preferred Reporting Items for Systematic Reviews and Meta-Analyses Extension for Scoping Reviews
RQ: research question
