# Supplementary material for: Home Visits as a Primary Health Care Strategy During COVID-19: Protocol for a Scoping Review
Source: JMIR Res Protoc. 2026 Aug 10;15:e97947. doi: 10.2196/97947 (PMC13457179; doi:10.2196/97947)
Supplement: Supplementary file 1 [file resprot-v15-e97947-s001.pdf]

Supplementary Material S2. Details of the Boolean search string used for each database.

MEDLINE/PubMed® via interface of the National Library of Medicine® (NLM®)

Blocks and returns	Search strings
#1 (Return: ‹n_1›)	("home visit*"[tiab] OR "house call*"[tiab] OR "home care service*"[tiab] OR "home health care"[tiab] OR "home nursing"[tiab] OR "home-based care"[tiab] OR "domiciliary visit*"[tiab] OR "domiciliary care"[tiab] OR "home-based primary care"[tiab] OR "community home visiting"[tiab] OR "household visit*"[tiab] OR "home follow-up"[tiab] OR "in-home care"[tiab] OR "in-home health service*"[tiab] OR "outreach visit*"[tiab] OR "family health strategy"[tiab] OR "remote patient monitoring"[tiab] OR "house calls"[MeSH] OR "home care services"[MeSH] OR "home nursing"[MeSH])
#2 (Return: ‹n_2›)	("COVID-19"[tiab] OR "COVID 19”[tiab] OR “COVID19”[tiab] OR “COVID-2019”[tiab] OR “SARS-CoV-2”[tiab] OR “SARS CoV 2”[tiab] OR “SARS-CoV2”[tiab] OR “2019-nCoV”[tiab] OR “nCoV-2019”[tiab] OR “SARS coronavirus 2”[tiab] OR “severe acute respiratory syndrome coronavirus 2”[tiab] OR “novel coronavirus”[tiab] OR “coronavirus infection*”[tiab] OR “coronavirus pandemic”[tiab] OR “coronavirus disease”[tiab] OR “COVID-19"[MeSH] OR "SARS-CoV-2"[MeSH] OR "coronavirus infections"[MeSH])
#3 (Return: ‹n_3›)	(#1) AND (#2)

Search refinement:

Publication years: ≥ January 1, 2020

Return after refinement:

‹*N*› studies in the test carried out in ‹*date*›.

Note: MeSH is the acronym for Medical Subject Headings; [tiab] is the shorthand for [Title/Abstract] in PubMed searches. A pilot return test was conducted on June 22, 2026, to assess the feasibility of the systematic review, resulting in 1,660 studies retrieved from PubMed.

Scopus™ via native interface

Blocks and returns	Search strings
#1 (Return: ‹n_1›)	TITLE-ABS-KEY("home visit*" OR "house call*" OR "home care service*" OR "home health care" OR "home nursing" OR "home-based care" OR "domiciliary visit*" OR "domiciliary care" OR "home-based primary care" OR "community home visiting" OR "household visit*" OR "home follow-up" OR "in-home care" OR "in-home health service*" OR "outreach visit*" OR "family health strategy" OR "remote patient monitoring")
#2 (Return: ‹n_2›)	TITLE-ABS-KEY("COVID-19" OR "COVID 19" OR "COVID19" OR "COVID-2019" OR "SARS-CoV-2" OR "SARS CoV 2" OR "SARS-CoV2" OR "2019-nCoV" OR "nCoV-2019" OR "SARS coronavirus 2" OR "severe acute respiratory syndrome coronavirus 2" OR "novel coronavirus" OR "coronavirus infection*" OR "coronavirus pandemic" OR "coronavirus disease")
#3 (Return: ‹n_3›)	(#1) AND (#2)

Search refinement:

Publication years: ≥ January 1, 2020
Document type: Article
Source type: Journal
Publication stage: Final

Return after refinement:

‹*N*› studies in the test carried out in ‹*date*›.

Note: TITLE-ABS-KEY expression indicates that the search was conducted simultaneously in the title, abstract, and keywords fields of the documents indexed in Scopus. A pilot return test was conducted on June 22, 2026, to assess the feasibility of the systematic review, resulting in 1,684 studies retrieved from the Scopus database.

Web of Science™ Core Collection via native interface

Blocks and returns	Search strings
#1 (Return: ‹n_1›)	TS=("home visit*" OR "house call*" OR "home care service*" OR "home health care" OR "home nursing" OR "home-based care" OR "domiciliary visit*" OR "domiciliary care" OR "home-based primary care" OR "community home visiting" OR "household visit*" OR "home follow-up" OR "in-home care" OR "in-home health service*" OR "outreach visit*" OR "family health strategy" OR "remote patient monitoring")
#2 (Return: ‹n_2›)	TS=("COVID-19" OR "COVID 19" OR "COVID19" OR "COVID-2019" OR "SARS-CoV-2" OR "SARS CoV 2" OR "SARS-CoV2" OR "2019-nCoV" OR "nCoV-2019" OR "SARS coronavirus 2" OR "severe acute respiratory syndrome coronavirus 2" OR "novel coronavirus" OR "coronavirus infection*" OR "coronavirus pandemic" OR "coronavirus disease")
#3 (Return: ‹n_3›)	(#1) AND (#2)

Search refinement:

Publication years: ≥ January 1, 2020
Document types: Article

Return after refinement:

‹N› studies in the test carried out in *‹date›*.

Note: TS indicates a search in the Topic field of Web of Science, covering the title, abstract, and keywords. A pilot return test was conducted on June 22, 2026, to assess the feasibility of the systematic review, resulting in 1,117 studies from the Web of Science database.

Embase™ via native interface

Blocks and returns	Search strings
#1 (Return: ‹n_1›)	('home visit*':ti,ab,kw OR 'house call*':ti,ab,kw OR 'home care service*':ti,ab,kw OR 'home health care':ti,ab,kw OR 'home nursing':ti,ab,kw OR 'home-based care':ti,ab,kw OR 'domiciliary visit*':ti,ab,kw OR 'domiciliary care':ti,ab,kw OR 'home-based primary care':ti,ab,kw OR 'community home visiting':ti,ab,kw OR 'household visit*':ti,ab,kw OR 'home follow-up':ti,ab,kw OR 'in-home care':ti,ab,kw OR 'in-home health service*':ti,ab,kw OR 'outreach visit*':ti,ab,kw OR 'family health strategy':ti,ab,kw OR 'remote patient monitoring':ti,ab,kw OR 'home visit'/exp OR 'house call'/exp OR 'home care'/exp OR 'home health care'/exp OR 'home nursing'/exp')
#2 (Return: ‹n_2›)	('covid-19':ti,ab,kw OR 'covid 19':ti,ab,kw OR 'covid19':ti,ab,kw OR 'covid-2019':ti,ab,kw OR 'sars-cov-2':ti,ab,kw OR 'sars cov 2':ti,ab,kw OR 'sars-cov2':ti,ab,kw OR '2019-ncov':ti,ab,kw OR 'ncov-2019':ti,ab,kw OR 'sars coronavirus 2':ti,ab,kw OR 'severe acute respiratory syndrome coronavirus 2':ti,ab,kw OR 'novel coronavirus':ti,ab,kw OR 'coronavirus infection*':ti,ab,kw OR 'coronavirus pandemic':ti,ab,kw OR 'coronavirus disease':ti,ab,kw OR 'covid-19'/exp OR 'sars-cov-2'/exp OR 'coronavirus infection'/exp OR 'covid-19 pandemic'/exp)
#3 (Return: ‹n_3›)	(#1) AND (#2)

Search refinement:

Publication years: ≥ January 1, 2020
Publication type: ≥ Article

Return after refinement:

‹N› studies in the test carried out in *‹date›*.

Note: The search was conducted in the title (ti), abstract (ab), and author keywords (kw) fields of records indexed in Embase. The '/exp' operator indicates the explosion of an Emtree descriptor, retrieving the term and all of its more specific terms in the hierarchy. A pilot return test was conducted on June 22, 2026, to assess the feasibility of the systematic review, resulting in 2,917 studies retrieved from the Embase database.

Cochrane Library® via native interface

Blocks and returns	Search strings
#1<br>(Return: ‹n_1›)	("home visit*" OR "house call*" OR "home care service*" OR "home health care" OR "home nursing" OR "home-based care" OR "domiciliary visit*" OR "domiciliary care" OR "home-based primary care" OR "community home visiting" OR "household visit*" OR "home follow-up" OR "in-home care" OR "in-home health service*" OR "outreach visit*" OR "family health strategy" OR "remote patient monitoring") in Title Abstract Keyword
#2<br>(Return: ‹n_2›)	("COVID-19" OR "COVID 19" OR "COVID19" OR "COVID-2019" OR "SARS-CoV-2" OR "SARS CoV 2" OR "SARS-CoV2" OR "2019-nCoV" OR "nCoV-2019" OR "SARS coronavirus 2" OR "severe acute respiratory syndrome coronavirus 2" OR "novel coronavirus" OR "coronavirus infection*" OR "coronavirus pandemic" OR "coronavirus disease") in Title Abstract Keyword
#3<br>(Return: ‹n_3›)	(#1) AND (#2)
	**Search refinement**:<br>Publication years: ≥ January 1, 2020<br>Publication type: Trials<br>**Return after refinement**:<br>‹*N*› studies in the test carried out in ‹*date*›.

Note: The search was conducted in the title, abstract, and keywords fields of the documents indexed in the Cochrane Library. A pilot return test was conducted on June 22, 2026, to assess the feasibility of the systematic review, resulting in 211 studies retrieved from the Cochrane Library database.

Literatura Latino-Americana e do Caribe em Ciências da Saúde (*LILACS*) via *Biblioteca Virtual em Saúde* (*BVS*) interface

Blocks and returns	Search strings
#1<br>(Return: ‹n_1›)	("home visit*" OR "house call*" OR "home care service*" OR "home health care" OR "home nursing" OR "home-based care" OR "domiciliary visit*" OR "domiciliary care" OR "home-based primary care" OR "community home visiting" OR "household visit*" OR "home follow-up" OR "in-home care" OR "in-home health service*" OR "outreach visit*" OR "family health strategy" OR "remote patient monitoring")
#2<br>(Return: ‹n_2›)	("COVID-19" OR "COVID 19" OR "COVID19" OR "COVID-2019" OR "SARS-CoV-2" OR "SARS CoV 2" OR "SARS-CoV2" OR "2019-nCoV" OR "nCoV-2019" OR "SARS coronavirus 2" OR "severe acute respiratory syndrome coronavirus 2" OR "novel coronavirus" OR "coronavirus infection*" OR "coronavirus pandemic" OR "coronavirus disease")
#3<br>(Return: ‹n_3›)	(#1) AND (#2)
	**Search refinement**:<br>Publication years: ≥ January 1, 2020<br>Document types: Not reviews<br>**Return after refinement**:<br>‹*N*› studies in the test carried out in ‹*date*›.

Note: The search was conducted in the title, abstract, and keywords fields of the documents indexed in the *LILACS* via *BVS* interface. A pilot return test was conducted on June 22, 2026, to assess the feasibility of the systematic review, resulting in 175 studies retrieved from the *LILACS* database.

Cumulative Index to Nursing and Allied Health Literature (CINAHL®) via EBSCOhost interface

Blocks and returns	Search strings
#1 (Return: ‹n_1›)	XB ("home visit*" OR "house call*" OR "home care service*" OR "home health care" OR "home nursing" OR "home-based care" OR "domiciliary visit*" OR "domiciliary care" OR "home-based primary care" OR "community home visiting" OR "household visit*" OR "home follow-up" OR "in-home care" OR "in-home health service*" OR "outreach visit*" OR "family health strategy" OR "remote patient monitoring") OR MW ("home visit*" OR "house call*" OR "home care service*" OR "home health care" OR "home nursing" OR "home-based care" OR "domiciliary visit*" OR "domiciliary care" OR "home-based primary care" OR "community home visiting" OR "household visit*" OR "home follow-up" OR "in-home care" OR "in-home health service*" OR "outreach visit*" OR "family health strategy" OR "remote patient monitoring")
#2 (Return: ‹n_2›)	XB ("COVID-19" OR "COVID 19" OR "COVID19" OR "COVID-2019" OR "SARS-CoV-2" OR "SARS CoV 2" OR "SARS-CoV2" OR "2019-nCoV" OR "nCoV-2019" OR "SARS coronavirus 2" OR "severe acute respiratory syndrome coronavirus 2" OR "novel coronavirus" OR "coronavirus infection*" OR "coronavirus pandemic" OR "coronavirus disease") OR MW ("COVID-19" OR "COVID 19" OR "COVID19" OR "COVID-2019" OR "SARS-CoV-2" OR "SARS CoV 2" OR "SARS-CoV2" OR "2019-nCoV" OR "nCoV-2019" OR "SARS coronavirus 2" OR "severe acute respiratory syndrome coronavirus 2" OR "novel coronavirus" OR "coronavirus infection*" OR "coronavirus pandemic" OR "coronavirus disease")
#3 (Return: ‹n_3›)	(#1) AND (#2)

Search refinement:

Publication Years: ≥ January 1, 2020
Publication type: Academic journal
Peer-reviewed study: Yes

Return after refinement:

‹*N*› studies in the test carried out in ‹*date*›.

Note: XB indicates that the search was conducted in the Title and Abstract fields, while MW refers to major subject headings in CINAHL. A pilot return test was conducted on June 22, 2026, to assess the feasibility of the systematic review, resulting in 1,139 studies retrieved from the CINAHL database.
