# Supplementary material for: Home Visits as a Primary Health Care Strategy During COVID-19: Protocol for a Scoping Review
Source: JMIR Res Protoc. 2026 Aug 10;15:e97947. doi: 10.2196/97947 (PMC13457179; doi:10.2196/97947)
Supplement: Supplementary file 2 [file resprot-v15-e97947-s002.pdf]

Supplemental Material S4. Return tests for the terms selected for the mandatory primary search pathway.

Block	Terms (title, abstract, keywords)	Database						
		PubMed	Scopus	Web of Science	Embase	Cochrane	LILACS	CINAHL
	"home visit*"	12,843	18,204	14,273	20,201	5,235	660	11,716
	"house call*"	801	4,952	934	18,442	820	454	623
	"home care service*"	3,363	40,384	3,642	1,046	2,970	966	1,797
	"home health care"	3,378	7,362	3,590	4,113	379	1,011	30,988
	"home nursing"	1,840	9,702	1,219	4,277	794	1,189	4,610
	"home-based care"	1,799	2,548	2,122	2,177	254	30	982
	"domiciliary visit*"	146	228	154	276	21	21	72
	"domiciliary care"	402	668	462	569	47	916	227
	"home-based primary care"	345	385	519	603	18	–	279
	"community home visiting"	1	6	4	8	4	–	1
	"household visit*"	474	657	492	719	188	7	142
	"home follow-up"	153	276	242	411	121	12	117
	"in-home care"	3,039	4,181	2,856	3,488	241	252	2,493
	"in-home health service*"	36	49	34	47	3	–	20
	"outreach visit*"	382	430	381	539	198	–	189
	"family health strategy"	1,065	2,103	1,703	1,222	52	6,512	1,766
	"remote patient monitoring"	1,633	6,353	2,623	2,414	241	22	853
	▶ "house calls"[MeSH]	4,500	–	–	–	–	–	–
	▶ "home care services"[MeSH]	54,271	–	–	–	–	–	–
	▶ "home nursing"[MeSH]	9,603	–	–	–	–	–	–
	▶ 'home visit'/exp	–	–	–	8,417	–	–	–
	▶ 'house call'/exp	–	–	–	8,417	–	–	–
	▶ 'home care'/exp	–	–	–	106,973	–	–	–
	▶ 'home health care'/exp	–	–	–	106,973	–	–	–
	▶ 'home nursing'/exp	–	–	–	106,973	–	–	–
#1	(t_1 OR t_2 OR … OR t_n)	**75,446**	**84,513**	**32,974**	**125,822**	**9,883**	**9,003**	**50,384**

Block	Terms (title, abstract, keywords)	Database						
		**PubMed**	**Scopus**	**Web of Science**	**Embase**	**Cochrane**	**LILACS**	**CINAHL**
	"COVID-19"	442,438	765,165	630,410	523,241	24,155	23,729	175,337
	"COVID 19"	442,438	765,165	630,410	523,241	24,155	23,729	2,174
	"COVID19"	418,198	8,783	10,181	517,508	772	23,728	894
	"COVID-2019"	533	948	688	692	43	42	76
	"SARS-CoV-2"	163,301	290,991	176,192	194,829	8,995	18,538	22,105
	"SARS CoV 2"	163,301	290,991	176,192	194,802	8,995	18,538	220
	"SARS-CoV2"	5,599	6,895	7,117	14,390	485	18,538	692
	"2019-nCoV"	2,243	4,442	2,591	2,729	287	17,405	302
	"nCoV-2019"	79	120	89	100	3	17,174	4
	"SARS coronavirus 2"	492	627	488	557	46	17,169	22
	"severe acute respiratory syndrome coronavirus 2"	43,114	155,050	42,151	44,438	2,501	17,122	5,789
	"novel coronavirus"	14,210	20,671	15,409	15,929	941	17,258	2,818
	"coronavirus infection*"	5,578	57,481	7,429	8,821	1,532	21,606	2,945
	"coronavirus pandemic"	4,301	11,070	8,095	4,623	123	16,985	1,467
	"coronavirus disease"	90,466	409,860	97,080	93,319	10,961	17,551	19,176
	▶ "COVID-19"[MeSH]	314,073	–	–	–	–	–	–
	▶ "SARS-CoV-2"[MeSH]	210,007	–	–	–	–	–	–
	▶ "coronavirus infections"[MeSH]	327,024	–	–	–	–	–	–
	▶ 'covid-19'/exp	–	–	–	521,499	–	–	–
	▶ 'sars-cov-2'/exp	–	–	–	153,945	–	–	–
	▶ 'coronavirus infection'/exp	–	–	–	543,694	–	–	–
	▶ 'covid-19 pandemic'/exp	–	–	–	201,319	–	–	–
**#2**	(t_1 OR t_2 OR … OR t_n)	**515,512**	**852,821**	**689,922**	**660,306**	**26,033**	**24,718**	**186,315**
**#3**	(#1) AND (#2)	**1,660**	**2,447**	**1,365**	**5,354**	**211**	**229**	**1,311**

Note: The symbol "▶" denotes MeSH terms followed by the suffix [MeSH]. The symbol "▶" denotes Emtree terms followed by the suffix /exp. The return tests for the search terms were conducted on June 22, 2026. *N* = 12,577 publications retrieved.
