# Supplementary material for: Home Visits as a Primary Health Care Strategy During COVID-19: Protocol for a Scoping Review
Source: JMIR Res Protoc. 2026 Aug 10;15:e97947. doi: 10.2196/97947 (PMC13457179; doi:10.2196/97947)
Supplement: Supplementary file 3 [file resprot-v15-e97947-s003.pdf]

Supplementary Material S3. Data extraction summary form of the studies included in this scoping review.

(A) Reference (Author, Year)	(B) Country/ Study center/ Health System	(C) Implementation month and year	(D) Locally reported pandemic milestones	(E) Assigned Pandemic phase	(F) Study objective	(G) Methodological design	(H) Population/ Participants	(I) Provider configuration	(J) Purpose of visits	(K) Delivery modality and level of digital integration	(L) Procedures/ Actions performed	(M) Safety protocols/ PPE	(N) Targeting logic	(O) Results and impacts	(P) Challenges/ Barriers	(Q) Facilitators/ Success factors	(R) Identified knowledge gaps	(S) Limitations	(T) Recommendations	(U) Funding and conflicts of interest
As an original peer-reviewed publication	Country, region, or city; PHC model (e.g., Brazil – Family Health Strategy)	Reported implementation month/year or period (do not infer missing dates)	Reported local waves, restrictions, vaccination, testing, variants, or service restoration	Phase reported or assigned using predefined rules; otherwise, "Not assigned."	State the primary aim of the study	Qualitative, quantitative, mixed, case study, cross-sectional study, etc.	Who was attended to or accompanied during the visits (e.g., elderly, pregnant women, chronic patients, vulnerable families)	How professionals were involved in home visits (e.g., single-discipline, multidisciplinary, interprofessional, outreach teams, etc.)	Main purpose of such clinical monitoring, surveillance, health education, psychosocial support, etc.	The interventions were exclusively in-person or hybrid (describe digital technologies used, and level of digital integration)	What was done during the visit (e.g., measurement, physical examination, guidance, referrals, etc.)	Measures to reduce transmission risk specify	Criteria or operational logic used to prioritize the visits (multiple categories may apply; more than one may be recorded)	Main findings such as clinical, social, organizational, or epidemiological	Difficulties reported such as lack of PPE, overload, fear, logistics, etc.	Elements that favored implementation, such as community support, clear protocols, prior experience, etc.	Conclusions about what still needs to be further studied, according to the authors	Summarize limitations that may affect the results	Practical, operational, or policy suggestions (e.g., expand telemonitoring)	As stated
Silva et al. (2021) [*]	Brazil; rural municipality; Family Health Strategy	April–September 2020	● Municipal mobility restrictions were introduced in March 2020<br>● Local community transmission increased in April 2020<br>● Vaccination was not yet available during implementation; routine consultations remained restricted throughout most of the reported period	Early response phase<br>(assigned on the basis of increasing local transmission, mobility restrictions, limited testing capacity, and absence of vaccine availability)	To describe how home visits were reorganized to monitor older adults in isolation	Qualitative study (semi-structured interviews)	Older adults living at home (n=32 households)	Nurse-community health worker dyad operating within a Family Health Strategy team	Clinical and medication adherence monitoring	● Hybrid model combining scheduled in-person home visits with weekly telephone check-ins for symptom monitoring and medication follow-up.<br>● Low (telephone calls were used for scheduling and for follow-up, but no video consultation or digital monitoring platform was reported.	● Symptom checklist<br>● BP check<br>● Medication delivery<br>● Counseling	N95 masks, gloves, surface disinfection, and outdoor consultations when possible	Proactive risk-stratified continuity care prioritizing older adults with chronic diseases, mobility limitations, social vulnerability, or difficulty accessing the primary care facility	Improved treatment adherence and reduced hospitalizations	Early PPE shortages and staff overload	Strong pre-existing bonds between the team and community	Need long-term impact evaluation	● Small sample size.<br>● Lack of longer-term evaluation<br>● Self-reported experiences, which may introduce recall bias.<br>● The rapidly changing pandemic context prevented the longitudinal follow-up needed to evaluate long-term impacts of home visit adaptations.	Develop standardized crisis-response home visiting protocols	● Municipal public funding.<br>● Authors declared no conflicts of interest.

Note: * Hypothetical example data.
